# Communities organizing to promote equity: engaging local communities in public health responses to health inequities exacerbated by COVID-19–protocol paper

**DOI:** 10.3389/fpubh.2024.1369777

**Published:** 2024-04-24

**Authors:** Christina M. Pacheco, Kristina M. Bridges, Edward F. Ellerbeck, Elizabeth Ablah, K. Allen Greiner, Yvonnes Chen, Vicki Collie-Akers, Mariana Ramírez, Joseph W. LeMaster, Kevin Sykes, Daniel J. Parente, Erin Corriveau, Antonio Miras Neira, Angela Scott, Kara E. Knapp, Jennifer Woodward, Sarah Finocchario-Kessler, COPE Team, Harshdeep Acharya, Clarissa Carrillo, Tatiana Darby, Jody Hoener, Allison Honn, Nadine Long, Mary Ricketts

**Affiliations:** ^1^Department of Family Medicine and Community Health, University of Kansas Medical Center, Kansas City, KS, United States; ^2^Department of Population Health, University of Kansas Medical Center, Kansas City, KS, United States; ^3^Department of Population Health, University of Kansas School of Medicine-Wichita, Wichita, KS, United States; ^4^School of Journalism and Mass Communications, University of Kansas, Lawrence, KS, United States; ^5^Department of Otolaryngology-Head and Neck Surgery, University of Kansas Medical Center, Kansas City, KS, United States; ^6^Health and Wellness Center, Baylor Scott and White Health, Dallas, TX, United States

**Keywords:** heath equity, rural, COVID-19, community health workers, community coalitions, public health, social determinants of health

## Abstract

**Background:**

The COVID-19 pandemic has disproportionately impacted rural and under-resourced urban communities in Kansas. The state’s response to COVID-19 has relied on a highly decentralized and underfunded public health system, with 100 local health departments in the state, few of which had prior experience engaging local community coalitions in a coordinated response to a public health crisis.

**Methods:**

To improve the capacity for local community-driven responses to COVID-19 and other public health needs, the University of Kansas Medical Center, in partnership with the Kansas Department of Health and Environment, will launch Communities Organizing to Promote Equity (COPE) in 20 counties across Kansas. COPE will establish Local Health Equity Action Teams (LHEATs), coalitions comprised of community members and service providers, who work with COPE-hired community health workers (CHWs) recruited to represent the diversity of the communities they serve. CHWs in each county are tasked with addressing unmet social needs of residents and supporting their county’s LHEAT. LHEATs are charged with implementing strategies to improve social determinants of health in their county. Monthly, LHEATs and CHWs from all 20 counties will come together as part of a learning collaborative to share strategies, foster innovation, and engage in peer problem-solving. These efforts will be supported by a multilevel communications strategy that will increase awareness of COPE activities and resources at the local level and successes across the state. Our mixed methods evaluation design will assess the processes and impact of COPE activities as well as barriers and facilitators to implementation using aspects of both the Consolidated Framework for Implementation Research (CFIR) and Reach, Effectiveness, Adoption, Implementation and Maintenance (RE-AIM) models.

**Discussion:**

This protocol is designed to expand community capacity to strategically partner with local public health and social service partners to prioritize and implement health equity efforts. COPE intentionally engages historically resilient communities and those living in underserved rural areas to inform pragmatic strategies to improve health equity.

## 1 Introduction

The COVID-19 pandemic spotlighted the stark health inequities experienced by historically resilient populations, such as American Indian and Alaska Native, Black/African American, Hispanic/Latino, Asian, Native Hawaiian, other Pacific Islander peoples, as well as rural populations (1). Many of these populations experienced higher burdens of COVID-19 infection, hospitalization, and death (2). Areas with high unemployment and uninsured residents had fewer COVID-19 testing or vaccination options than more affluent areas and these residents experienced significant barriers to accessing quality healthcare services (3). In 2021, the COVID-19 death rate for Kansas was 103.1 per 100,000 people, the 24th highest in the country (4).Although Kansas has urban areas with racially, culturally, and linguistically diverse populations, most of the state is rural (5). Many rural and frontier counties in Kansas rely heavily on a patchwork of federally qualified health centers (FQHCs), rural health clinics, and local health departments for routine and preventive care (6). Local health departments have been historically underfunded and understaffed nationally, but this is particularly true in Kansas, which currently ranks 48th in the nation in per capita public health funding (7). Residents in rural communities, many of whom lack basic access to healthcare and social services, are more likely to experience inequities, including poverty, poorer health, and limited transportation services compared to their urban counterparts (8–10). Residents in under-resourced urban communities experience additional barriers to good health, e.g., the legacy of redlining policies and practices, gentrification, and exposure to crime and violence (11). The resultant health inequities are a product of social, economic, environmental, and structural disparities. These disparities exacerbate differences in health outcomes within and between communities (12) and make residents more vulnerable to severe adverse outcomes or death due to COVID-19 (13).

In recognition of persistent health inequities, the National Institutes of Health (NIH) launched the Rapid Acceleration of Diagnostics - Underserved Populations (RADx-Up) initiative in 2020 to rapidly scale up access to COVID-19 testing among populations experiencing inequities in COVID-19 morbidity and mortality (14). Other national institutions, including the Centers for Disease Control and Prevention (CDC), increased investment in state health departments to improve health equity through addressing health disparities exacerbated by COVID-19 (15). The Triple Aim of Health Equity includes approaches that (1) strengthen the capacity of communities to create healthy futures, (2) allow residents to generate ideas, contribute to decision-making, and have mutual accountability for outcomes, and (3) appreciate that this process requires time, flexibility, persistence, humility, and significant investment (16). Consistent with this approach, Communities Organizing to Promote Equity (COPE) will uniquely invest in communities to improve health equity through equitable community engagement, including trust building, open communication, shared decision-making, resource-sharing, mutual benefit, and bi-directional learning (17–19). This paper describes the design, objectives, and implementation strategies for COPE to strengthen community capacity, elicit the experiences and recommendations of community residents with lived experience, and partner with public health and social service organizations to address local health equity issues in Kansas.

## 2 Methods/design

### 2.1 COPE protocol description

COPE is an academic-community partnership that began June 2021, with funding support through May 2024 via the Kansas Department of Health and Environment through CDC funds to address health disparities exacerbated by the COVID-19 pandemic (CDC-RFAata-OT21-2103: National Initiative to Address COVID-19 Health Disparities Among Populations at High-Risk and Underserved, Including Racial and Ethnic Minority Populations and Rural Communities) (20). This funding allows COPE to focus on the broad range of health disparities related to and exacerbated by COVID-19 and empower communities to identify their priority health equity foci. We grounded our approach in Human-Centered Design (21), which offers strategies to partner with community stakeholders, understand their experiences, and co-design interventions. By using this approach, we centralized the needs and priorities of populations experiencing health inequities focused on capacity building and co-creating equity-aware strategies.

COPE will significantly expand a novel approach to community engagement called Local Health Equity Action Teams (LHEATs), that was employed in a NIH COVID-19-related study in Kansas in 2020 (RADx-Up, UL1TR002366-04S3) (22, 23). The COPE protocol was designed by investigators from the RADX-UP project, community liaisons, and community health workers. Evaluation data collected from the community partners in RADx-Up was utilized to guide modifications employed in the COPE protocol (e.g., ensuring community residents were members of the LHEATs, employing CHWs to assist with the work of the LHEAT, etc.). LHEATs include representation from local health departments, community-based organizations, social service organizations, FQHCs, rural health clinics, and community residents with lived experience who have experienced barriers to health and can voice perspectives of historically resilient populations. We hope these efforts will result in diverse LHEAT membership that will include: racial and ethnic populations, people residing in urban, rural, and frontier geographies, people experiencing houselessness, people who are refugees, those who are uninsured, those of low-socioeconomic status, and lesbian, gay, bisexual, transgender and/or gender expansive, queer and/or questioning, intersex, asexual, and two-spirit (LGBTQIA2S+) (24, 25) individuals.

Given the critical role of community health workers (CHW) in addressing health equity and to ensure LHEATs will include members with dedicated time to support outreach and implementation, COPE will employ a minimum of two to a maximum of three CHWs per county, based on population and staff availability. This will result in a workforce of more than 50 CHWs across the state.

**Figure 1:**
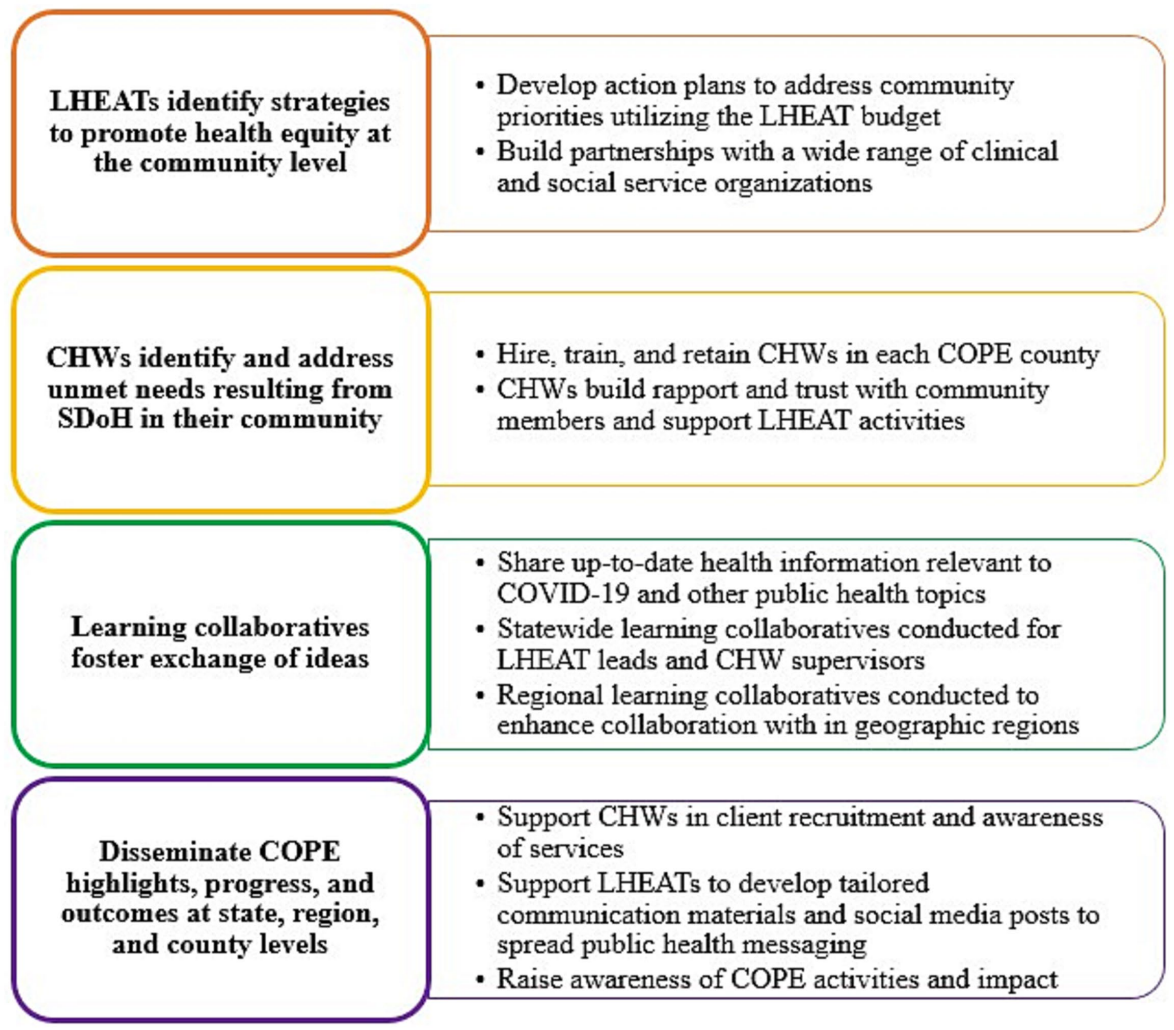
COPE objectives.

COPE has four parallel main objectives: (1) establish or grow LHEATs equipped to develop and implement strategies to enhance health equity in their county, (2) hire and train community health workers CHWs that will support LHEAT activities and address the needs of at-risk members of their community, (3) institute learning collaboratives to foster exchange of ideas across communities, and (4) engage in multilevel dissemination of COPE resources, health equity messages, and outcomes (Figure 1). The overarching goal for COPE is to build infrastructure for public health and future pandemic response that elevates community engagement to prevent disproportionate impact on historically resilient populations.

### 2.2 Setting

**Figure 2:**
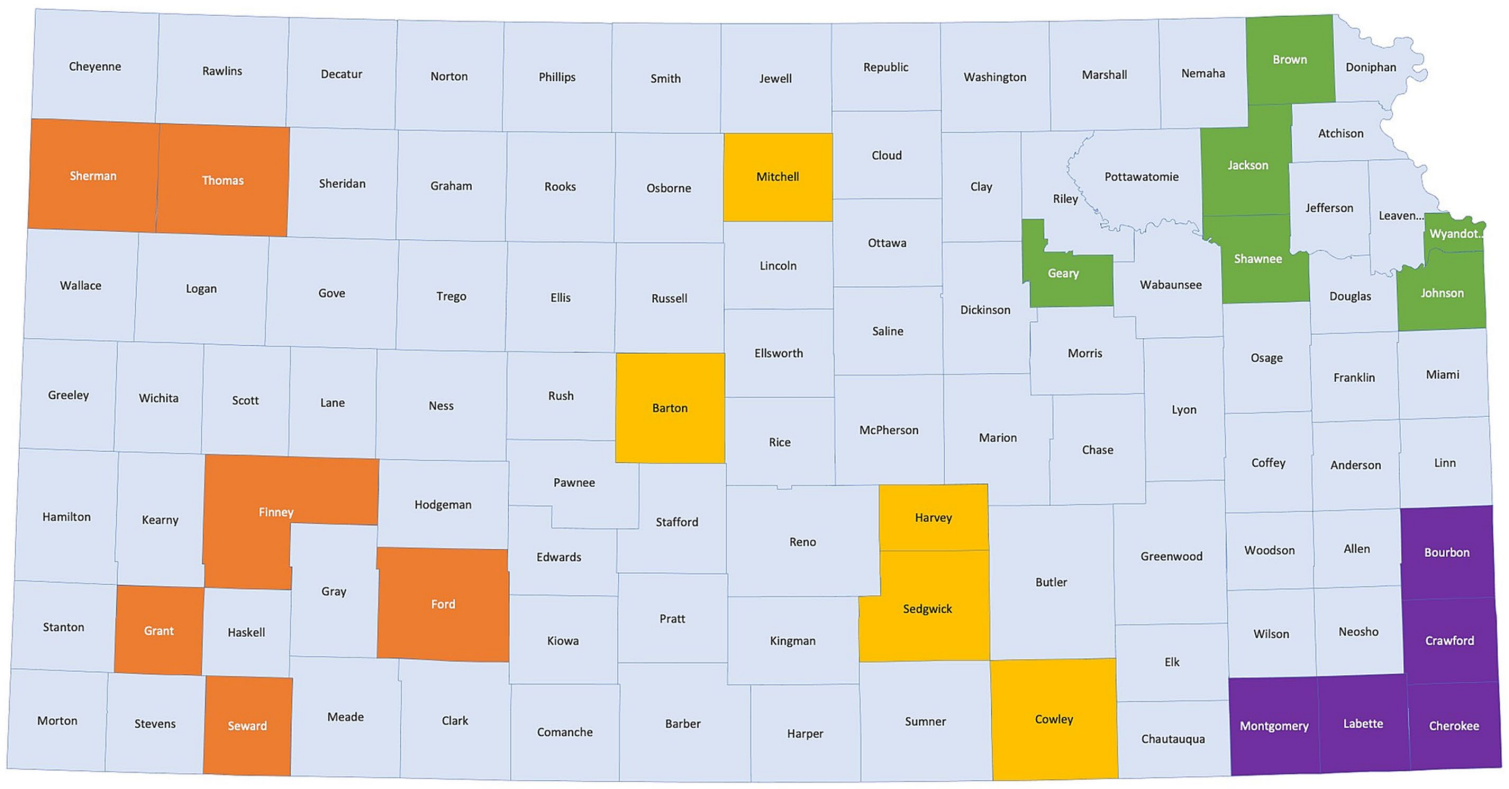
Map of Kansas with COPE counties highlighted by regions.

To balance engagement across the state, we will include five counties in each of four regions of the state (west, central, northeast, and southeast). Counties will be selected and prioritized using multiple criteria to determine which counties are experiencing the most significant health disparities. This includes using: the Kansas Public Health rankings, data from the Social Vulnerability Index and the Area Deprivation Index (26, 27), rates of COVID-19 infection, testing and vaccination, and existing/emerging relationships developed with local community leaders and organizations (Figure 2).

### 2.3 Support structure

**Figure 3:**
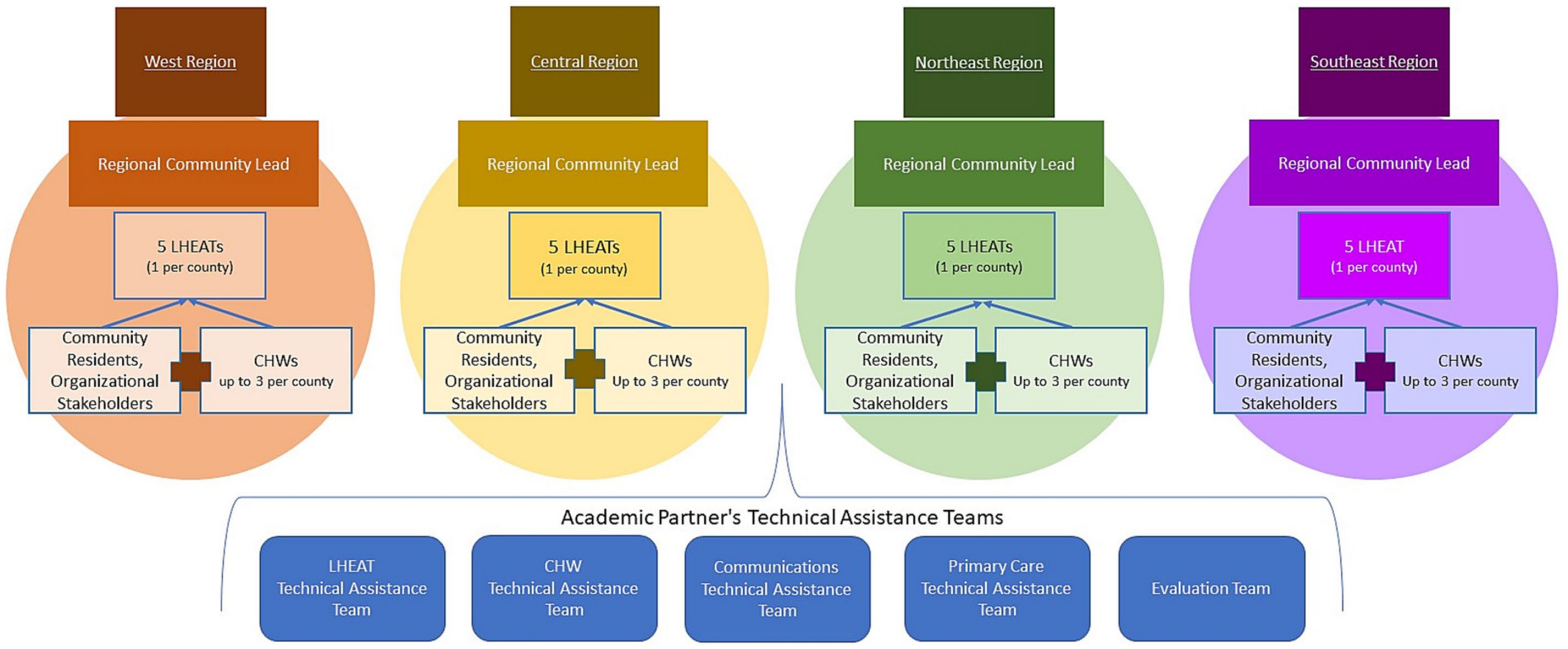
COPE Technical Assistance Structure.

Several technical assistance teams will support COPE across Kansas (Figure 3). The LHEAT technical assistance team will be tasked with providing training and contributing ongoing technical support regarding strategies to address social impediments to health. The CHW technical assistance team will be tasked with hiring, onboarding, and training CHWs across the state and providing monthly performance-based feedback. The Communications team will be responsible for increasing COPE’s visibility, strengthening collaborative relationships, customizing the design of promotional materials for a range of populations, managing the website, and disseminating COPE highlights. At the regional level, on-the-ground project managers called regional community leads (RCL), will be hired for each of the four regions. Each RCL will be selected for their experience with community engagement, geographic location, and ability to provide on-the-ground support and assistance to the five LHEATs and 10 to 15 CHWs in their region. RCLs will assist with LHEAT formation, CHW hiring, attend LHEAT meetings, and support strategic planning and problem-solving for their regional teams. Additionally, a cadre of primary care physicians, several of whom are county health officers, will aid in initial partnership formation between LHEATs, FQHCs, and public health departments across the state. They will also provide health information relevant to COVID-19 (e.g., case rates and vaccination information) and other health topics (e.g., Mpox, influenza, RSV, etc.) to LHEATs and CHWs. An evaluation team, composed of mixed methods scientists, will evaluate all aspects of COPE.

### 2.4 Local health equity action teams

LHEATs will design innovative strategies for addressing and removing impediments to health and wellbeing in their counties (28). The LHEAT model utilized in RADx-Up Kansas (22, 23) demonstrated promise in diverse urban and rural communities whose populations had some of the highest COVID-19 case rates across Kansas (22, 23). Thus, LHEATs will be created and expanded with an intent of social inclusion (i.e., members will be strategically recruited to include those most impacted by social impediments to health and those working to address impediments). Although membership will vary by county, we will intentionally recruit individuals from the following key populations: People of Color, low-income, uninsured, rural residents, older adults (65+), individuals with disabilities, LGBTQIA2S+, multilingual, refugees, immigrants, and limited English proficiency. Those who are otherwise at-risk for COVID-19, such as those who work in close confines (e.g., meat processing plants) or have poor living conditions (e.g., Kansans who are unhoused) will also be populations of focus (28).

One LHEAT member from each county will be designated the “LHEAT lead” to serve as the facilitator for the group. In addition to leadership and facilitation training, the LHEAT lead will receive training on upstr‑eam (systemic or chronic), downstream (immediate), and community power-building approaches (29, 30) to assist with idea generation on addressing their communities’ needs. LHEAT leads will receive a monthly stipend of $500 to support their role.

**Figure 4:**
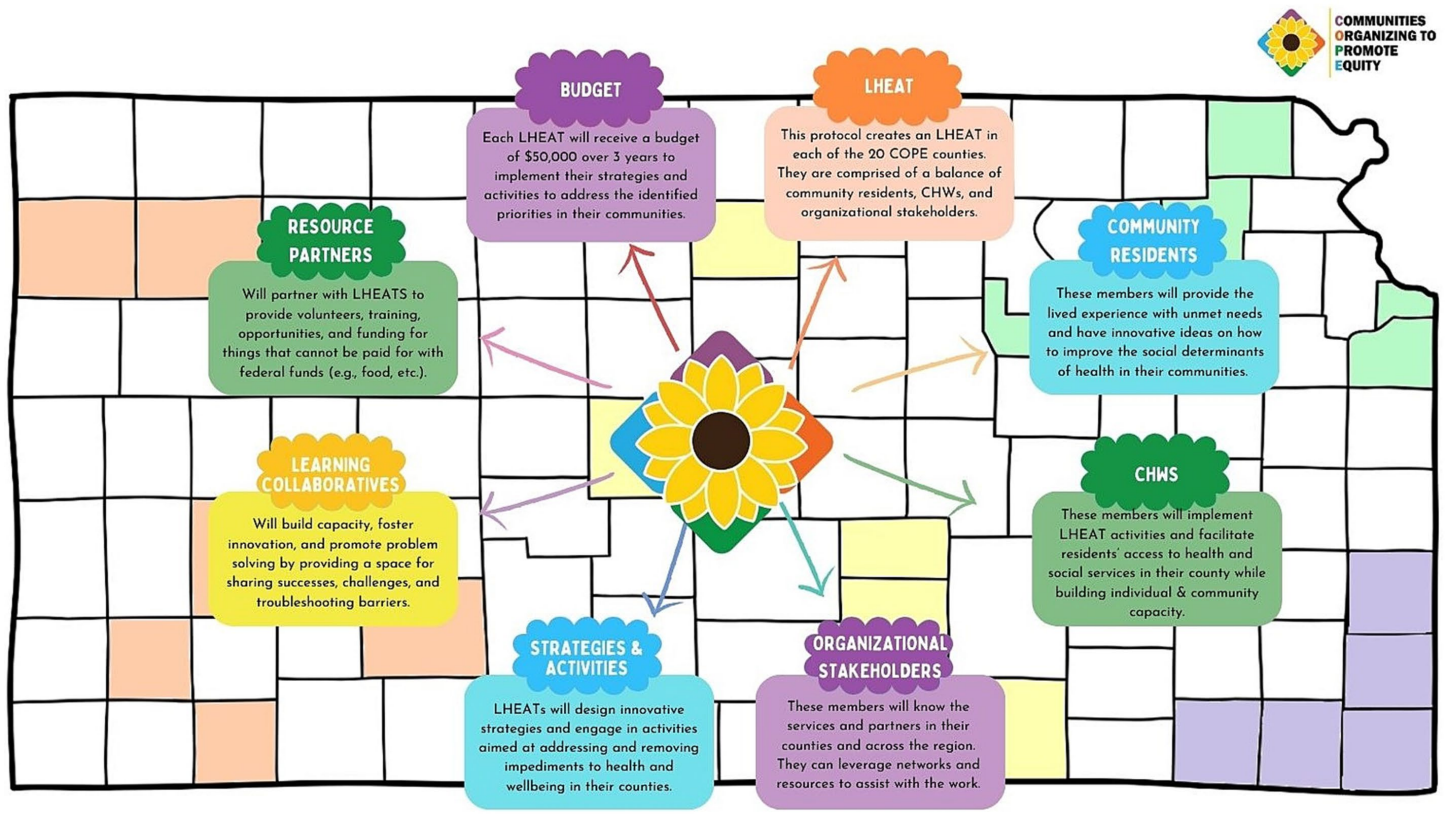
Graphic representation of COPE elements.

LHEATs will be charged with balancing their membership among (1) community residents who will bring lived experiences, (2) organizational representatives (e.g., social service agencies, local health departments, etc.), and (3) COPE community health workers (CHWs). To help remove barriers to participation, LHEAT members will receive a $40 gift card for each meeting they attend. Moreover, each LHEAT will have a budget of $50,000 to implement the strategies and activities upon which they collectively agree over the three-year period. LHEATs will utilize an action planning document (30-day action plan) that aids in identifying: (1) why their topic is a priority activity, (2) who are the intended beneficiaries, (3) what are the action steps necessary for implementation, (4) who will complete the action steps, (5) what are the expected outcomes/impacts, and (6) a draft budget (Figure 4).

### 2.5 Community health workers

COPE CHWs will be tasked with two primary functions—implementing LHEAT activities and facilitating access to health and social services to address unmet social needs for individuals and families in their county (31). Active, involved, and trusted community members will be sought after to fulfill the role of CHW. CHWs will build individual and community capacity by increasing health knowledge and self-sufficiency through outreach, community education, informal counseling, social support, and advocacy (31). For COPE, community members will be hired, trained, and credentialed as CHWs and integrated into community organizations, FQHCs, rural health clinics, and/or local health departments in each COPE county. Each county will be afforded up to three full-time CHWs, with the option to combine part-and full-time CHW positions. CHW training will include COPE-specific training focused on daily responsibilities and database training to access, enter, and review client data. Training focuses on the core competencies of the CHW role, will be provided by a state approved CHW training program, and will qualify them for certification by the state of Kansas. One CHW in each county will be trained as a supervisor to meet with their teams weekly to support team building, engage in conflict management, provide supervision, and clarify key metrics of COPE.

The CHWs will foster cross-sectoral partnerships, identify resource partners to assist with LHEAT initiatives, support public health initiatives, help clients access healthcare, and make referrals to social service organizations (e.g., housing, transportation, food). COPE CHWs will be expected to spend approximately 70% of their time in the community, in contrast to the more traditional placement of CHWs in clinics or organizations. CHWs will receive client referrals through partnerships they build in their counties, individual requests for assistance, and communication and educational materials. To capture COPE CHWs’ range of activities, we will develop a database to track the number and type of community partnerships created, outreach events or activities conducted, and number of clients enrolled and served (32).

### 2.6 Learning collaboratives

Learning collaboratives will be designed to build regional capacity, foster innovation, and promote problem solving. The LHEAT technical assistance team will support a monthly statewide learning collaborative for the 20 LHEAT leads to share their work and foster innovation and advocacy. During these meetings, LHEAT Leads will share their successes and challenges and seek assistance to troubleshoot barriers. Guest presenters will be invited to inspire continuous innovation in priority areas (e.g., linkage to care, transportation, affordable childcare services). The CHW technical assistance team will support a statewide learning collaborative for all CHWs every other month to share case management, lessons learned, and innovations to overcome common barriers. Additionally, RCLs will lead monthly regional learning collaboratives for the LHEAT Leads and CHWs in the five counties comprising each of their respective regions in the state: northeast, southeast, central, and west (Figure 2).

### 2.7 Multilevel communications and dissemination

The COPE communications team aligns the design and dissemination of communication activities with the COPE objectives. Guided by an overarching strategy of cultivating and maintaining relationships with internal and external stakeholders, the team will provide support in four key tactical areas: website, e-newsletter, storytelling, and tailored communication support. The team will maintain a website (33) that includes information about LHEATs, CHWs, and news/reports from COPE. A referral form with a quick response (QR) code will be consistently embedded in communication promotional materials, allowing potential clients to contact CHWs for assistance directly. The team will disseminate a monthly e-newsletter that highlights successes of LHEATs and CHWs in addressing community needs and will spotlight notable achievements from COPE partners (e.g., community-and faith-based organizations).

The communications team will also respond to requests from LHEATs and CHWs to design promotional materials for community events and aid in social media use. The team will create COPE-branded, county-specific Facebook pages, provide training on social media content creation and analytics for all LHEATs, and supplies public health information for LHEAT Facebook pages.

### 2.8 Evaluation

The COPE evaluation plan will be grounded in implementation science and will examine COPE processes, outcomes, and determinants of success (34). Evaluation design, qualitative and quantitative data collection, analysis, and outcomes assessment are structured around the Consolidated Framework for Implementation Research (CFIR) and Reach, Effectiveness, Adoption, Implementation, and Maintenance (RE-AIM) models to examine contextual determinants of the implementation process and assess the impact of COPE strategies and activities (34).

The mixed methods evaluation design will be used to determine the extent to which the planned program activities are completed (process indicators), assess the extent to which these activities lead to expected short-and long-term outcomes (outcome measures), identify barriers and facilitators to achieving COPE objectives (qualitative implementation evaluation), and leverage the evaluation to drive continuous quality improvement (Table 1).

**Table 1:** COPE protocol process indicators and outcomes.

COPE objective	Process indicators	Short-term outcomes	Long-term outcomes
LHEATs Identify strategies to promote health equity at the community level	-LHEATs established in each county with strategic and inclusive membership including historically resilient groups^1,3^ -LHEATs identify priority needs^2,3,4^ -LHEATs implement budgets to address priority needs^2,3^ -LHEATs partner with community orgs to address SDOH^2,4^	LHEAT activities address priority areas^2,3,4^ -Partner organizations engaged in efforts^2,3,4^ Community needs and available resources are characterized^2,4^ -Improved access to COVID-19 vaccination and testing^2,4^ -Community events address priority community needs^2^	LHEATs are sustained after funding ceases. Local and state health departments leverage the LHEAT to inform public health practice. Increased capacity of LHEAT members to organize and impact health equity locally
CHWs identify and address social determinants of health in their community	CHWs hired, trained, and certified in each county^4,5,8^ -CHWs equipped to provide education and promotion on COVID-19 vaccination & testing^4,5^ -CHWs are prepared to engage historically resilient groups^5^ -CHWs are active members of their respective LHEATs^2,3^	CHWs work with clients to assess needs, set goals and work to achieve goals^4^ -CHWs develop referral relationships with clinics and organizations^4^ -CHWs identify resources and act as liaisons between organizations and clients^4^ -Improved access to COVID-19 vaccination and testing^2,4^	Reports generated on value of investing in CHWs -Influence policies regarding reimbursement of CHW services at the institution and state-level -Improved referral networks and access to resources for addressing SDoH needs -CHW positions are maintained beyond COPE funding
Learning collaboratives foster exchange of ideas	LHEAT leads and CHWs participate in monthly learning collaboratives^6^ Regional teams (LHEATS & CHWs from 5 counties) participate in quarterly learning collaboratives	Satisfaction and perceived value of learning collaboratives^8,9^ Regional and cross-regional project collaboration^2,4,9^ Enhanced training on time-sensitive topics^6^	Sustained regional and cross-regional collaborations to address social determinants of health
Disseminate COPE engagement opportunities, progress, and outcomes at state, region, and county levels	Training provided to LHEATs to build local communication capacity^6^ COPE website, social media, and newsletter created and distributed^7^	LHEATs use social media pages to promote activities and public health messaging^7^ -Community partners included in regional and national presentations and publications^7^ Community utilization of media produced^2,7^	Communities better able to craft public health messaging -COPE presentations and publications build the evidence base for sustainable investment in local health networking and empowerment Increase in national support for community-based efforts

^1^LHEAT intake survey. ^2^30-Day action plans. ^3^LHEAT vital sign check-in. ^4^COPE Database, ^5^CHW demographics and training database. ^6^Learning Collaborative records. ^7^Dissemination indicators. ^8^CHW surveys. ^9^LHEAT surveys.

Process indicators and short-and long-term outcomes associated with each of the four COPE objectives are detailed in Table 1. Process metrics to identify needs for ongoing quality improvement will be evaluated quarterly. Short-term outcomes for COPE include establishing LHEATs, hiring and training CHWs in each of the 20 COPE counties, initiating monthly learning collaboratives, and creating and leveraging communication outlets. Intermediate outcomes for COPE include: (1) increased awareness of community needs and available resources, (2) the creation of a directory of partners and services available in each county, and (3) CHWs building relationships with clinics and community-based organizations for continued referral services after COPE.

Long-term outcomes for COPE include: (1) strengthening the role of communities in the public health response to COVID-19 and addressing social needs, (2) reducing COVID-19 related health disparities and improving outcomes for historically resilient populations, and (3) improving statewide and local health capacity and services for COVID-19 prevention and control.

#### 2.8.1 Data sources for process and outcome measures

Seven data sources will be used for process and outcome measures.

1. *LHEAT intake surveys* will be conducted at the time of onboarding to capture demographics, including social identities which have been historically excluded and marginalized beyond race/ethnicity. Members will indicate why they joined and what they hope to contribute.
2. *30-day action plans* will document how each LHEAT focuses health equity efforts and implements strategies (events, programs, and/or advocacy for policy, practice, systems, and/or environmental changes). LHEATs will indicate in their 30-day action plans whether their activities were upstream (addressing conditions creating SDoH), downstream (easing the negative impact of inequitable conditions), and/or LHEAT power building (capacity and influence in the community) and submit a budget and justification for proposed costs.
3. *LHEAT vital sign check-ins* will include brief monthly surveys to be completed by each LHEAT lead to capture data on LHEAT activities, barriers, partnerships, membership status, meetings, and communications.
4. *COPE database* will be a secure, HIPAA-compliant, comprehensive, electronic, cloud-based application designed for COPE based on input from experienced CHWs. The database will capture CHW client information (demographics, needs, goals), organizational partners (contact information, location, services offered), referrals to and from CHWs, and events (location, purpose, intended beneficiaries, attendance) (32).
5. *CHW demographics and training database* will capture data from employment and training records to collect the number of CHWs hired, content and amount of training completed, certification acquired, type and location of hosting organization.
6. *Learning collaborative records* will document attendance and topics addressed at the monthly state and regional learning collaborative meetings.
7. *Dissemination indicators* will include COPE website analytics (views and engaged sessions), monthly newsletter analytics, COPE and LHEAT Facebook metrics (reach, impressions, and engagements), number of promotional materials produced, number of digital stories produced and analytics (views), abstracts, and manuscripts.

#### 2.8.2 Qualitative evaluation

The COPE qualitative evaluation will be developed to identify barriers and facilitators to implementing COPE activities and attaining COPE objectives. We will conduct two rounds of interviews with CHWs, LHEAT leads, and LHEAT members. The first round will focus on early implementation and be conducted shortly after LHEAT formation. Round one interview questions will be guided by the CFIR model (35) and focus on four CFIR domains (e.g., outer setting, inner setting, intervention characteristics, and process of implementation). Round two interviews will focus on impact and be conducted approximately 6 months before the end of COPE. Round two interview questions will be based on the RE-AIM model (36) to probe the respondent’s perceptions about COPE activities’ reach, effectiveness, and future maintenance. In addition to COPE LHEAT members, representatives from partner organizations, and staff will be interviewed during the second round to understand lessons learned.

### 2.9 Sustainability

This protocol was developed with sustainability in mind. CHWs will be hired by FQHCs and placed in communities, with the hopes that these partners will be able to eventually bill for CHW services. LHEATs will also focus on power building strategies such as training community members on grant writing to sustain their work. Additionally, support staff will work with local health departments and governments to support/sustain LHEATs and their initiatives.

## 3 Discussion

Through a focus on community power building and the provision of technical assistance, COPE is designed to strengthen community capacity to elevate community-driven priorities and implementation strategies. Critical to our success, our social inclusion strategy will acknowledge the diversity in our communities and include individuals with a range of wisdom and worldviews in this work while establishing sustainable partnerships with local organizations serving those who encounter social impediments to health. COPE aims to build sustainable county, regional, and state-level equity infrastructure to improve Kansas’s public health capacity.

Often, attempts at community engagement offer a prescribed checklist of activities in which community members are “engaged.” However, COPE will intentionally build LHEATs to ensure that members, especially community residents and CHWs, are empowered to contribute to the decision-making and innovation processes, distribute resources and labor, and have shared accountability for the outcomes, successes, and learning opportunities. Public health practitioners that want to engage individuals who experience health inequities need to confront imbalances of power and create systems that support shared leadership (16). We believe this protocol will strengthen counties’ abilities to address the existing impediments to health and ensure the conditions are ripe to improve health equity.

Rather than using a clinic-based model of CHWs focused on extending clinical care from healthcare institutions into communities (37), the COPE CHWs will be embedded with community-based organizations, social service organizations, local health departments, and other non-traditional facilities, like local libraries or faith-based organizations, which make them more accessible to community members. CHWs will also serve as paid staff to ensure implementation of the LHEAT activities and initiatives.

Significant amounts of funding have focused on understanding and addressing health disparities, yet health disparities in the United States have not drastically improved. Many traditional approaches have failed to engage the people most familiar with the barriers and detriments of these disparities in understanding and combatting them (38). Rather than relying on evidence-based practice strategies or prescribed interventions, in which the evidence may not be created with the inclusion of minoritized communities, COPE will make the space for communities to generate evidence that is anchored in life experiences and community innovation. Furthermore, rather than just soliciting suggestions, COPE will allocate funds to each LHEAT to facilitate piloting LHEAT-driven strategies and activities to empower a greater sense of autonomy among LHEATs and generate data to support future grant applications.

Our protocol will have limitations. Although a significant strength of this protocol is allowing each county to customize elements of COPE (e.g., the number of CHWs, using an existing coalition as their LHEAT vs. recruiting from scratch), this variation will make it challenging to measure the impacts of the differing elements. Permitting different hiring strategies for CHWs, e.g., some CHWs will be hired directly by the academic partner and others directly by local organizations (i.e., FQHCs, community organizations), will add complexity to balancing protocol and employer priorities. The pace of the hiring and LHEAT formation processes will vary across counties and may result in staggered start times across the state. Another major limitation of the protocol is that substantial continued funding will be needed to sustain the community infrastructure that COPE will establish.

## 4 Conclusion

COPE has the potential to create unique opportunities to engage communities in launching community-driven strategies to promote health equity. Implementation across 20 geographically dispersed counties with widely varying demographics and resources will provide an opportunity for understanding the ability of the COPE model to adapt to local circumstances. COPE will provide an essential understanding of the impact of partnering with community residents, CHWs, and service organizations to impact social determinants of health and health inequities.

## Data availability statement

The original contributions presented in the study are included in the article/supplementary material, further inquiries can be directed to the corresponding author.

## Ethics statement

This study was approved by the Institutional Review Board at the University of Kansas Medical Center (IRB# STUDY00148455). Informed consent will be obtained from participants.

## Author contributions

CP: Project administration, Supervision, Writing – original draft, Writing – review & editing, Conceptualization, Methodology. KB: Writing – original draft, Writing – review & editing. EE: Funding acquisition, Investigation, Methodology, Project administration, Writing – review & editing, Writing – original draft. EA: Funding acquisition, Investigation, Methodology, Project administration, Writing – review & editing, Writing – original draft. KG: Conceptualization, Funding acquisition, Investigation, Project administration, Writing – review & editing. YC: Project administration, Resources, Writing – original draft, Writing – review & editing. VC-A: Writing – original draft, Writing – review & editing. MR: Conceptualization, Investigation, Project administration, Resources, Writing – review & editing. JL: Writing – review & editing, Funding acquisition, Project administration, Visualization. KS: Investigation, Resources, Writing – review & editing, Formal analysis, Methodology. DP: Writing – review & editing. EC: Writing – review & editing. AM: Writing – review & editing, Project administration, Resources. AS: Writing – review & editing, Project administration, Resources. KK: Writing – review & editing, Project administration, Resources. JW: Resources, Writing – review & editing. SF-K: Conceptualization, Data curation, Formal analysis, Funding acquisition, Investigation, Methodology, Project administration, Resources, Supervision, Validation, Visualization, Writing – original draft, Writing – review & editing.

## COPE Team

In addition to the listed authors, the COPE Team includes Harshdeep Acharya, MD^1,7^, Clarissa Carrillo, MPA^1^, Tatiana Darby, MPH^1,2^, Jody Hoener, MSW^8^, Allison Honn, MBA^3^, Nadine Long, MPA^1^, Mary Ricketts^9^.

1. Department of Family Medicine and Community Health, University of Kansas Medical Center, Kansas City, KS, United States
2. Department of Population Health, University of Kansas Medical Center, Kansas City, KS, United States
3. Department of Population Health, University of Kansas School of Medicine-Wichita, Wichita, Kansas, United States
4. School of Journalism and Mass Communications, University of Kansas, Lawrence, KS, United States
5. Department of Otolaryngology-Head and Neck Surgery, University of Kansas Medical Center, Kansas City, KS, United States
6. Health and Wellness Center, Baylor Scott & White Health, Dallas, TX, United States
7. Department of Internal Medicine, Saint Peter’s University Hospital, New Brunswick, NJ, United States
8. Healthy Bourbon County Community Action Team, Fort Scott, Kansas, United States
9. Turning Point Training & Development LLC, Overland Park, Kansas, United States
